# A Simple Screening and Optimization Bioprocess for Long-Chain Peptide Catalysts Applied to Asymmetric Aldol Reaction

**DOI:** 10.3390/molecules28196985

**Published:** 2023-10-09

**Authors:** Shulin Wang, Haidong Teng, Lan Wang, Pengcheng Li, Xinghao Yuan, Xi Sang, Jianping Wu, Lirong Yang, Gang Xu

**Affiliations:** College of Chemical and Biological Engineering, Zhejiang University, Hangzhou 310058, China

**Keywords:** peptide catalysis, asymmetric aldol reaction, fusion protein, simple bioprocess

## Abstract

Peptides have demonstrated their efficacy as catalysts in asymmetric aldol reactions. But the constraints inherent in chemical synthesis have imposed limitations on the viability of long-chain peptide catalysts. A noticeable dearth of tools has impeded the swift and effective screening of peptide catalysts using biological methods. To address this, we introduce a straightforward bioprocess for the screening of peptide catalysts for asymmetric aldol reactions. We synthesized several peptides through this method and obtained a 15-amino acid peptide. This peptide exhibited asymmetric aldol catalytic activity, achieving 77% ee in DMSO solvent and 63% ee with over an 80.8% yield in DMSO mixed with a pH 9.0 buffer solution. The successful application of our innovative approach not only represents an advancement but also paves the way for currently unexplored research avenues.

## 1. Introduction

Chiral alcohols play a significant role as fundamental components employed in the pharmaceutical, agrochemical, and fragrance industries and the fine chemical sector, among other areas [1,2]. The asymmetric aldol reaction plays a pivotal role in constructing the C-C bond of chiral alcohols in a single step [3]. A variety of catalysts have been reported in relation to the asymmetric aldol reaction, including metals [4], enzymes [5,6], catalytic antibodies [7,8], and small molecules (also known as organocatalysts) [9,10,11,12]. For instance, in 2007, Inoue reported that chiral bis(oxazolinyl)phenyl-rhodium complexes served as catalysts in the coupling of AgOTf for the direct aldol reaction of ketones and aromatic aldehydes, resulting in the formation of corresponding β-hydroxyketones with high *anti*-selectivity and high enantioselectivity with an up to 91% enantiomeric excess (ee) value. However, the use of metal catalysts presents limitations due to factors such as high cost, limited availability, challenging ligand design, and environmental concerns, making them less than ideal for certain applications [13]. Enzymes, owing to their promiscuity, have the potential to catalyze non-natural reactions. In 2013, Chen reported that trypsin could catalyze asymmetric aldol reactions; however, the selectivity and yield in these reactions were limited, with yields ranging from 7% to 60% and ee values ranging from 16% to 65% [14]. In 1999, List and colleagues discovered that the aldolase antibody 38C2 catalyzed the enantioselective aldol cyclodehydration of 4-substituted-2,6-heptanediones, resulting in the formation of enantiomerically enriched 5-substituted-3-methyl-2-cyclohexen-1-ones. After a 2d reaction, the yield exceeded 92%, while the selectivity was moderate [15]. In organic molecules capable of catalyzing aldol reactions, cinchona alkaloids, chiral thioureas, and amino acids have all been proven to be effective organocatalysts. In 2007, Zheng reported a highly enantioselective direct aldol reaction catalyzed by primary amines derived from cinchonine. This catalysis enabled clean reactions between aromatic aldehydes and various cyclic ketones, yielding *anti*-aldol adducts in an up to 99% yield, with good diastereoselectivities (up to 9:1) and excellent enantioselectivities (up to 99% ee) [16]. In 2012, Bastida designed a dual-function amine-thiourea catalyst for asymmetric aldol reactions, achieving selectivity and yields exceeding 90% [17]. List, while observing aldolase X-ray diffraction structures, combined the amino group of lysine residues and the acidic group of tryptophan residues involved in aldolase catalysis. He realized that to catalyze an Aldol reaction, a catalyst needs to possess both an amino group and a carboxylic acid group, i.e., the origin of non-metallic enzyme activity, which is relevant to the reaction mechanism. Coincidentally, proline has these attributes and offers structural stability, providing a rigid effect in protein structures. He therefore reported in 2000 that the yield was 68% and the selectivity was 76% when acetone and p-nitrobenzaldehyde were catalyzed by 30 mol% proline in DMSO [18]. Subsequently, he referred to proline as a “micro-aldolase”. In 2021, he was awarded the Nobel Prize in Chemistry for his contributions to the field of asymmetric organic catalysis.

In organocatalysts, peptide catalysts have been recognized as powerful and useful tools in the asymmetric field. However, current peptide catalysts for asymmetric aldol reactions are primarily limited to short peptides [19], peptides with N-terminal proline [20,21], or proline derivatives [22,23,24]. In 2013, Huang reported a series of valine dipeptide organocatalysts containing a primary amine group and two amide units. These catalysts were assessed in the direct asymmetric intermolecular aldol reaction between 4-nitrobenzaldehyde and cyclohexanone. Remarkably, when 2,4-dinitrophenol was included as an acidic additive, these catalyzed reactions involving various aldehydes and ketones produced the corresponding aldol products with moderate to high enantioselectivities (up to 95%) and diastereoselectivities (up to 99/1, *anti/syn*) in brine. This work represents a significant contribution to the field of organocatalysis for asymmetric aldol reactions catalyzed by short peptides [25]. In 2023, Wang et al. synthesized a heterogeneous catalyst, L-Pro-L-Pro-L-Phe-L-Phe-OMe, by combining an aromatic amino acid dipeptide with a proline dipeptide through solid-phase synthesis. Remarkably, L-Pro-L-Pro-L-Phe-L-Phe-OMe outperformed homogeneous small molecule catalysts in asymmetric aldol reactions, achieving high enantioselectivity (>97%), and it displayed excellent recyclability. This study represents a significant advance in asymmetric aldol reactions catalyzed by heterogeneous peptide catalysts with N-terminal proline [26]. In 2023, Al-Momani and colleagues synthesized hydroxyproline-based dipeptides by coupling Boc-protected *cis*- or *trans*-4-L-hydroxyproline with benzylated glycine and L-valine. These dipeptides, lacking a proton donor in the catalytic site, surprisingly exhibited good stereoselectivity favoring R-configured aldol products. These results were observed at moderate conversions over a 24 h period, marking a significant advancement in short-peptide catalysis [27]. In 2014, Bayat et al. designed and synthesized mimetic peptides inspired by promiscuous aldo-keto-reductase enzymes for use as multifunctional organocatalysts in direct asymmetric aldol reactions. Their work demonstrated that these peptides enabled the production of aldol products with impressive yields (up to 97%), exceptional diastereoselectivities (up to 99/1), and high enantioselectivities (>98%) under mild reaction conditions. Notably, they found that the presence of proline at the N-terminus of the peptide was crucial for achieving these favorable outcomes. This research highlights the potential of such mimetic peptides as effective catalysts in asymmetric aldol reactions [28]. In 2021, Peme reported the design and synthesis of mimetic peptides inspired by the catalytic active site of the fructose-1,6-bisphosphate aldolase enzyme, omitting proline at the N-terminus. These peptides exhibited catalytic activity in direct asymmetric aldol reactions. Although the yields of aldol products were relatively low (up to 44%), the peptides demonstrated excellent enantioselectivity (up to 93%) and moderate diastereoselectivity (65:35). Notably, when reacting acetone and 4-nitrobenzaldehyde, yields were as low as 10–20%, with selectivities ranging from 70% to 82%. In the presence of DMSO as a solvent, only trace amounts of the product were formed. This research highlights the potential of these peptides for enantioselective catalysis in aldol reactions [29].

**Figure 1:**
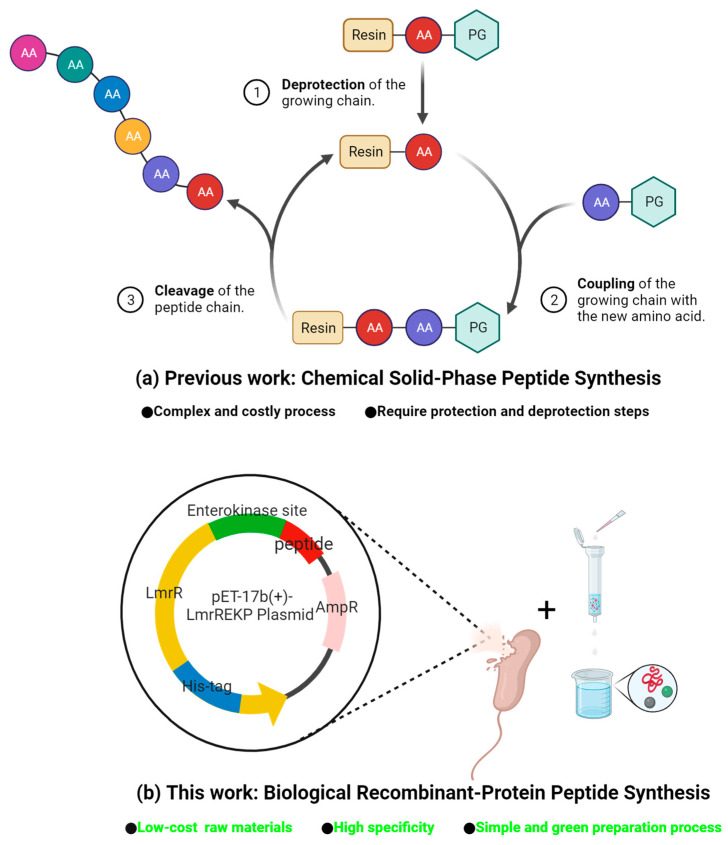
Comparison of chemical and biological peptide preparation methods.

While long-chain peptides are highly modular, structurally diverse, and easily accessible from nature’s chiral pool [30,31], they are simpler in structure compared to enzymes and possess the atom economy of organic small molecules [32]. So, they should have been an attractive candidate for discovering or designing new green catalysts [33,34,35]. However, long-chain peptide catalysts were deemed unnecessary to synthesize based on a comparison between tripeptides and tetrapeptides. The thorough exploration of the comprehensive catalytic capabilities of long-chain peptide catalysts has remained incomplete, as this investigation primarily commenced with proline-derived tripeptides, followed by the extension of the peptide chain using natural amino acids [36]. As we have described before, current peptide catalysts are usually prepared through chemical synthesis [37]. Traditional methods typically require an excess of amino acids, coupling reagents, and protective groups to achieve maximum conversion at each step, and these conditions do not meet the requirements of green chemistry. Furthermore, when dealing with longer-chain peptides, these challenges are exacerbated [38,39]. Due to the susceptibility of short peptides to degradation within living organisms, the production of peptide catalysts through biological methods is not common [40]. Moreover, the purification of a peptide requires preparative chromatography or gel column separation, heavily requiring the use of expensive organic solvents and complex purification procedures with high instrumentation dependency [41,42]. Hence, the imperative lies in advancing the biological methods of screening for catalysts of long-chain peptides, creating numerous unexplored opportunities awaiting discovery through the curiosity of biochemists. Figure 1 shows the comparison of traditional chemical synthesis and genetic engineering strategies.

**Figure 2:**
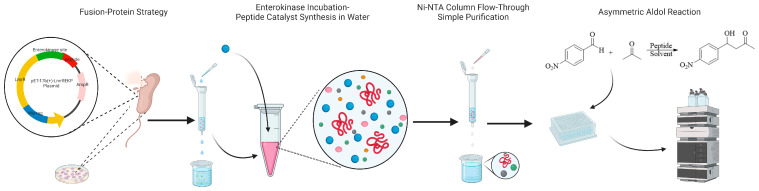
The whole process of the preparation and screening of peptide catalysts in asymmetric aldol reactions.

To address these challenges, in this study, we present a simple bioprocess for the preparation and screening of relatively long-chain peptide catalysts used in asymmetric aldol reactions. Our strategy revolves around the fusion of different peptides onto a protein scaffold using genetic engineering. The fusion-protein-based peptide libraries can be easily accessed through single- or multiple-point mutations, altering the amino acid sequences [43]. Moreover, the use of enterokinase under aqueous conditions allows for the controlled release of the peptide catalysts, and the ingenious utilization of a Ni-NTA gravity column effectively eliminates His-tagged large proteins. This enables the creation of diverse peptide libraries with minimal use of organic reagents and eliminates the need for one-by-one amino acid coupling and extensive purification steps. We successfully employed this approach to screen and identify several peptides that can effectively catalyze asymmetric aldol reactions. Figure 2 shows the whole process of the preparation and screening of peptide catalysts in asymmetric aldol reactions. A peptide with a non-proline N-terminus and 15 amino acids was able to catalyze an asymmetric aldol reaction, yielding a 77% ee value in DMSO and a 63% ee value with a yield of 80.8% when DMSO was combined with a pH 9.0 buffer solution. This application of our innovative approach in screening and optimizing peptide catalysts provides an effective tool for exploring new research possibilities in peptide-catalyzed asymmetric reactions.

## 2. Results and Discussion

Traditionally, TEV protease is used for specific cleavage to release the peptides in fusion proteins that contain specific cleavage sites for peptides. Because the recognition site of TEV protease is between glutamine and glycine or serine and a glycine would be left in the N-terminal of the peptide, the obtained peptide and the target peptide have different sequences. As a result, we decided to use enterokinase, a specific protease that does not leave behind any redundant amino acids after hydrolyzing. So, we firstly employed genetic engineering to construct a fusion protein, LmrREKP, by incorporating the specific recognition sequence for enterokinase (DDDDK) and a peptide into the LmrR protein scaffold. This modification allowed for efficient cleavage by enterokinase, facilitating the separation of the fusion protein and the peptide. To address the issue of the fusion protein’s inability to utilize a His-tag for adsorption on the Ni-NTA gravity column, we introduced a new His-tag at the N-terminus of the original LmrR protein and removed a His-tag from the C-terminus. This might have been possible due to the fact that an N-terminal His-tag is more likely to be exposed to the surface of an LmrR protein than a C-terminal His-tag, thus affecting affinity adsorption. Additionally, the fusion protein provided a solution to the short half-life and low stability issues typically associated with peptides expressed separately.

**Figure 3:**
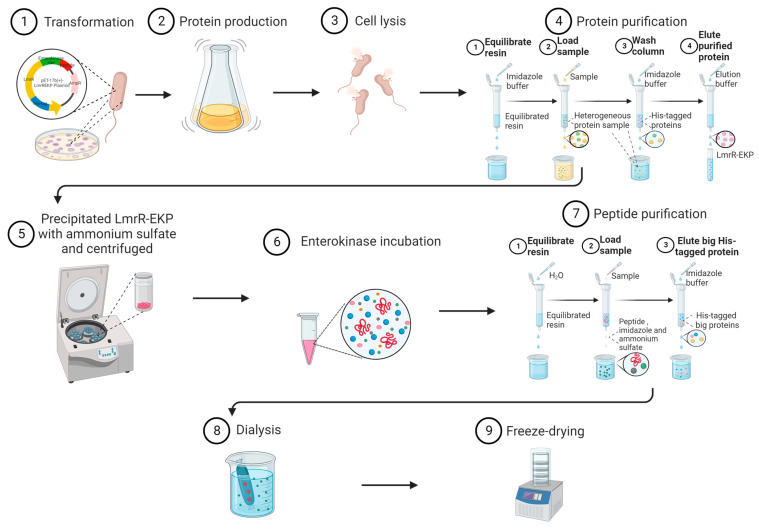
General process of peptide preparation and purification. Red represents peptide, pink spheres represent LmrREKP, blue spheres represent enterokinase, orange spheres represent LmrR, yellow and light-green spheres represent unobjective proteins, dark-green spheres represent imidazole, and gray spheres represent ammonium sulfate.

We transformed the resulting expression plasmid, pET-17b(+)-LmrREKP, into *E. coli* BL21(DE3) cells and expressed LmrREKP using the conventional pET expression system. Next, we purified LmrREKP with the His-tag at the N-terminus using Ni-NTA affinity chromatography and precipitated the protein with ammonium sulfate. The fusion protein was then resuspended in distilled water, and a portion of the sample was analyzed using SDS-PAGE. To release the peptide, we incubated the fusion protein solution with His-tagged enterokinase overnight and then removed the large His-tagged protein through Ni-NTA affinity chromatography. The filtrate contained the peptide along with ammonium sulfate and imidazole. After overnight dialysis (or not), we freeze-dried the peptide to obtain it in a powdered form. Figure 3 shows the general process of peptide preparation and purification.

**Figure 4:**
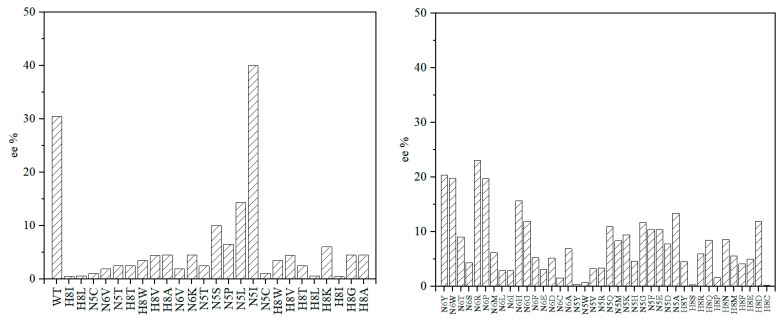
Establishment of the peptide library through single-point saturation mutations. The ee on the left figure was calculated using the dominant *R* configuration, and that on the right is reversed.

In the aldol reaction, the peptide powder demonstrated selectivity. We initially synthesized several peptides using this method and selected MYSFNNDHSEGAHPR, which exhibited the best selectivity, for constructing the peptide library. The sequences of these peptides were obtained via the hydrolysis of wild-type *Bn*LTA with trypsin. (There may be single or multiple amino acid differences in this case.) We selected peptides that contained amino acid sites considered to greatly influence selectivity in LTA and peptides that are highly alkaline because aldol reactions could be conducted without selectivity under alkaline conditions. The catalytic effects of these peptides are shown in Table 1. To create the peptide library, we utilized pET-17b(+)-LmrREKP2 as a template for single-point mutations, altering the primary structure of the peptide. Through the previous analysis of L-threonine aldolase results, it can be seen that N5, N6, and H8 on MYSFNNDHSEGAHPR have a great influence on the catalytic selectivity of aldolase, so we selected these three sites for saturation mutation [44]. The catalytic effects of peptides obtained by site mutation are shown in Figure 4 and Table 2.

**Figure 5:**
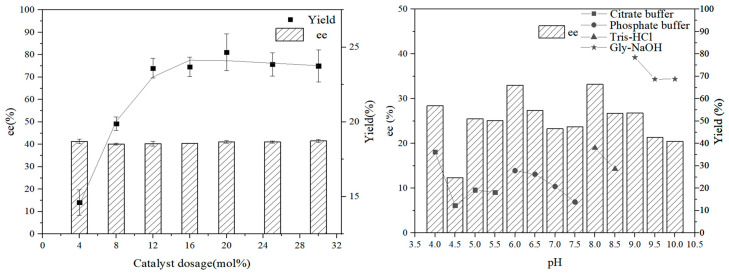
Effect of peptide dosage and pH in different buffer solutions on the asymmetric aldol reaction between acetone and p-nitrobenzaldehyde.

We validated selectivity using the chemically synthesized peptide MYSFINDHSEGAHPR and an optimized reaction system. The solvent, temperature, peptide dosage and pH of buffer solution are optimized and displayed respectively in Table 3 and Table 4 and Figure 5. In a pH 9.0 buffer solution, the same reaction resulted in an ee value of 34% and a yield of 87.2%.

In addition to testing the peptide’s catalytic performance in aqueous solutions, we also induced reactions in organic solvents. Based on the previous impact of solvents on the aldol reaction, we opted for using DMSO as a solvent and optimized the reaction system accordingly. We first optimized temperature, and the effect of temperature on peptide catalysis showed a trade-off effect. The higher the temperature, the higher yield, but the lower selectivity. Therefore, we chose 37 °C as a compromise temperature with relatively good yield and selectivity. After that, the water content was optimized, and the effect was best when the water content was 10 μL. We also measured the added amounts of acetone and peptide. As the added amount of acetone increased, the yield increased; when the added amount of peptide was more than 20 mol%, the reaction effect was not particularly improved. Considering the influence of different pH buffer solutions in an aqueous solvent on the reaction, a pH 9.0 buffer solution was selected to replace water for the reaction. In the DMSO solvent, the aldol reaction between acetone and p-nitrobenzaldehyde achieved 63% ee and a 47.2% yield, and when the reaction carried on for more than one week, the yield reached 80.8% without losses in ee (Table 5 Entry 13).

The successful development of the peptide library and the green and efficient purification of the fusion protein demonstrated the feasibility and reliability of our method. The use of enterokinase for the controlled release of the desired peptide further confirmed the functionality of the fusion protein. The peptide catalysts obtained through this approach demonstrated promising potential in asymmetric aldol reactions.

## 3. Materials and Methods

### 3.1. General Materials

Unless otherwise noted, all chemicals and reagents were obtained from commercial suppliers (Sinopharm Chemical Reagent (Shanghai, China), Sigma-Aldrich (St. Louis, MO, USA), Aladdin (Bay City, MI, USA), Macklin (Shanghai, China), Yuanye (Shanghai, China), and Sangon (Shanghai, China)) and used without further purification. All colored figures were created on BioRender.com (accessed on 15 August 2023).

### 3.2. Construction of LmrR Fusion Protein Plasmid Containing Peptide and Enterokinase Sites

LB medium used was composed of 10 g·L^−1^ of NaCl, 10 g·L^−1^ of peptone, and 5 g·L^−1^ of yeast extract at a natural pH. The LB agar medium was obtained by adding 2% agar into LB medium. *E. coli* Trans1-T1(TransGen Biotech, Beijing, China) was used as the host to construct, maintain, and amplify plasmids. *E. coli* strains were cultured in LB medium with 100 μg·mL^−1^ of amp. The PrimeSTAR Max DNA polymerase (TaKaRa, Dalian, China) was used for PCR amplifications. *Dpn*I was purchased from Takara (Dalian, China). Enterokinase was purchased from Yuanye (Shanghai, China). The enzymes described above were used according to the manufacturer’s specifications. All plasmid constructs were purified with plasmid extraction kits (TransGen Biotech, China) and verified via sequencing (Youkang, Hangzhou).

pET-17b(+) was used as the cloning and expression vector for all proteins described in this study. The pET-17b(+) plasmid came from our laboratory, in which it had been stored in beforehand, and its original sequence can be found in Supporting Information S1. All gene sequences were optimized through the website https://www.novopro.cn/tools/codon-optimization.html (accessed on 15 August 2023), and primers were designed on the website https://crm.vazyme.com/cetool/multipoint.html (accessed on 15 August 2023). All primers (synthesized by Youkang, Hangzhou) used in this study are listed in Supporting Information S2.

We first used EKF, EKR, PF, and PR primers to introduce the desired enterokinase cleavage site and peptide sequence into the pET-17b(+) plasmid, through PCR, between the stop codon and LmrR.

The PCR program was set as follows: 2.5 min at 98 °C, followed by 30 cycles of 10 s at 98 °C, 30 s at 60 °C, and 1.5 min at 72 °C, with a final extension step of 2.5 min at 72 °C. After verification via 1% agarose gel electrophoresis, the restriction endonuclease *Dpn*I (1 μL) was added into the amplified products (10 μL) and incubated at 37 °C for 1 h to digest the template plasmid DNA. After purification, the digested products were transferred into *E. coli* BL21(DE3) via heat shock and resuscitated at 37 °C for 1 h in a liquid medium. The resuscitated bacterial solution was plated onto LB agar medium containing Amp (100 μg·mL^−1^) and cultured overnight at 37 °C.

Subsequently, we removed the C-terminal His-tag using StopF and StopR primers and added an N-terminal His-tag with NHisF and NHisR primers. By employing this method, we successfully generated fusion protein plasmids containing both the peptide of interest and the enterokinase cleavage site, providing a versatile tool for the expression and purification of fusion proteins. Furthermore, this allowed for straightforward modification of the peptide’s primary structure via conventional mutagenesis methods. The final fusion protein was named LmrREKP. The complete sequence of the pET-17b(+)-LmrREKP plasmid containing the LmrREKP2 fusion gene is provided in Supporting Information S3.

### 3.3. Preparation and Purification of Peptides and Establishment of Peptide Library

The pET-17b(+)-LmrREKP2 plasmid was used as a template for the single-point mutation of the target amino acid site via whole-plasmid PCR. The candidate sites were mutated into 19 other amino acids. The PCR program used is the same as that described in the preceding section. *E. coli* colonies transformed with mutant plasmids on the plate were selected and inoculated into 5 mL LB liquid medium containing 100 µg·mL^−1^ of Amp. The LB medium was subjected to incubation in a shaker at 37 °C and 220 rpm for a duration of 6–8 h. Subsequently, 2 mL of this LB medium was introduced into a 100 mL LB liquid medium supplemented with 100 µg·mL^−1^ of Amp. The left cells were verified via sequencing. This LB medium underwent further incubation in a shaker operating at 220 rpm and 37 °C for a period of 2.5–3 h. Following this, an induction step was initiated by adding 100 µL of 0.5 M IPTG to the solution, which was then subjected to incubation in a shaker at 18 °C and 220 rpm for 16–18 h. The induced expressed cells were collected in a 100 mL centrifuge tube and centrifuged at 4 °C at 8000 rpm for 15 min. The supernatant was poured out, and the cell pellet was resuspended in 10 mL of water. The resuspended cells were then subjected to ultrasound treatment in an ice bath at a power of 400 W, with an ultrasonic rupture duration of 3 s and an interval of 7 s. After sonication, cell debris was removed via centrifugation at 12,000 rpm at 4 °C for 15 min, and the supernatant containing the target protein was obtained.

The pET17b(+)-LmrREKP mutant protein was purified using Ni-NTA affinity chromatography, with a 6×His tag added to the N-terminus. Supernatant or soluble fraction was filtered through a 0.22 μm syringe filter and applied to the Ni-NTA resin in a gravity column. The imidazole solution was pre-adjusted to pH 7.0 with HCl and then diluted with water. The binding buffer (50 mM of imidazole, pH 7.0) was used to elute the unbound proteins, and the target proteins were eluted using elution buffer (500 mM of imidazole, pH 7.0). The protein elution, precipitated with ammonium sulfate, was collected via centrifugation and stored at −20 °C. The protein collected was dialyzed in water, and this process was performed again using SDS-PAGE for analysis. All purification steps were carried out at 0–4 °C.

During the previous stage, an enterokinase cleavage site was introduced between the LmrR and the peptide, enabling incubation with enterokinase at 37 °C. At this stage, the solution contained three large proteins (enterokinase, LmrR protein, and uncut LmrREKP protein), all with 6×His tags, while the peptide remained without a His-tag. To purify the peptide in an environmentally friendly manner, we chose to pass the mixture through a Ni-NTA gravity column after filtration through a filter membrane. The His-tagged large proteins were captured by Ni^2+^, while the flow-through contained the peptide along with ammonium sulfate and imidazole. After overnight dialysis and lyophilization, the peptide powder was obtained and stored at −20 °C.

### 3.4. Agarose Gel Electrophoresis Analysis

A 1 Kb DNA Ladder (Marker) was purchased from TransGen Biotech (Beijing, China). The sample was mixed with 9 μL of 10× DNA loading buffer, and agarose gel electrophoresis was performed at 220 V for approximately 25 min (Supporting Information S4).

### 3.5. SDS-PAGE Analysis

The supernatant (15 μL) was mixed with 5 μL of 4× Protein SDS-PAGE loading buffer and heated to 99 °C in a metal block for 10 min. The samples were then loaded onto the protein gel and separated at 150 V for approximately 1.5 h. Following electrophoresis, the gel was stained with coomassie brilliant-blue solution. Subsequently, the gel was immersed in a destaining solution composed of water, ethanol, and acetic acid. Gentle agitation was applied to ensure efficient destaining. The destaining process was visually monitored, and upon achieving the desired destaining level, the solution was periodically replaced. Depending on the gel thickness and staining intensity, the destaining process typically lasted for 30 min to several hours. After completing the distaining process, the gel was rinsed with water to remove any remaining traces of the destaining solution, at which point it was ready for further analysis (Supporting Information S5).

### 3.6. Chemical Synthesis of Peptides

We purchased the peptide synthesized by conventional solid-phase peptide synthesis (SPPS) from Jiangsu Ji Tai Peptide Industry Science and Technology Co., Ltd. (Yancheng, China). The purity of the peptide was higher than 90%.

### 3.7. Chiral HPLC Analysis

After reaction, a 10 μL portion of the reaction solution was added to a mixture of n-hexane and isopropyl alcohol, each with a volume of 300 μL, and filtered through a 0.22 μm syringe filter. This was conducted to remove any particulate matter or impurities from the sample. The selectivity of the reaction was then determined via chiral HPLC. The HPLC was equipped with a CHIRALPAK^®^ IB N-5 column (4.6 × 250 mm, 5 μm) and a UV detector operated at 254 nm. Mobile phase consisted of a mixture of n-hexane and isopropyl alcohol in a ratio of 90:10 or 95:5 (*v*/*v*). The flow rate was set at 1.2 mL·min^−1^, and the temperature was maintained at 40 °C (Supporting Information S7). The enantiomeric excess of the product (ee) was calculated as follows:

$$\mathrm{ee}=\frac{\left(c_{\mathrm{major}}-{\;c}_{\mathrm{minor}}\right)}{\left(c_{\mathrm{major}}{+c}_{\mathrm{minor}}\right)}\times 100\%$$

### 3.8. Preparation of Aldol Reaction Standard Product Racemer and NMR Analysis

The reaction system: 10 mmol of aldehyde, a slight excess of ketone, and 4 mmol each of D and L-proline. The mixture was allowed to react for one day and then extracted with 25 mL of DCM. The organic layer was dried over MgSO_4_ and evaporated, and the crude product was further purified via column chromatography using n-hexane/ethyl acetate (7:1) as an eluent. The purified products of Aldol reaction were dissolved in CDCl_3_ for NMR analysis. ^1^H NMR spectra were recorded on BRUKER AVANCE NEO/500 MHz spectrometers. CDCl_3_ was purchased from J&K and used as a solvent (Supporting Information S8). All yields in this paper are isolated yields or were estimated using HPLC (external standard method).

## 4. Conclusions

In this study, we successfully developed a green and efficient method for preparing and isolating peptide catalysts applied to asymmetric aldol reaction based on a microbial protein expression system. By fusing specific protease cleavage sites and different peptides onto a protein scaffold, we achieved the controlled release of peptide catalysts using enterokinase under aqueous conditions, bypassing the laborious manual amino-acid-coupling process. The use of enterokinase for the controlled release of peptides further confirmed the functionality of the fusion protein. Subsequently, the peptides were collected through Ni-NTA gravity column adsorption and directly used in an aldol reaction after freeze-drying, eliminating the need for toxic organic solvents and costly purification steps. Further expansion of the peptide library through single-point mutations altered the primary structure of the peptides, enhancing reaction selectivity. Using this approach, we obtained a peptide consisting of 15 amino acids and a non-proline N-terminus. This peptide could catalyze an asymmetric aldol reaction and yielded 77% ee in DMSO solvent and 63% ee with an over 80.8% yield in DMSO mixed with a pH 9.0 buffer solution. The successful establishment of the peptide library and the green and efficient purification of fusion proteins have demonstrated the feasibility and reliability of our method. Our method provides a novel approach for preparing, screening, and optimizing peptide catalysts and opens new research possibilities for exploring broader and deeper applications in asymmetric catalysis.

## Figures and Tables

**Table 1:** Peptide catalyst initial screening for asymmetric aldol reaction.

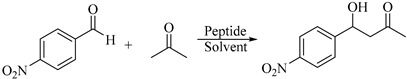			
Entry	Peptide Catalyst	ee%	Yield%
1	LVYISNSTER	7(R)	2.4
2	MYSFNNDHSEGAHPR	31(R)	5.9
3	GTIYSK	3(S)	39.2
4	GAMLAK	13(S)	55.6
5	GDNDLVLSDFPK	19(S)	9.4
6	GNYQFR	17(S)	13.0
7	GDHSAIR	25(S)	18.2

Reaction conditions: 0.05 mmol of p-nitrobenzaldehyde, 150 μL of solvent containing acetone/H_2_O 3/1 (*v*/*v*), and 4 mol% peptide were used for 5 days at room temperature while being agitated at 250 rpm in orbital shaker unless noted otherwise.

**Table 2:** The effect of different amino acids in peptides on the asymmetric aldol reaction.

Entry	Peptide Catalyst	ee%	Yield%
1	MYSFNNDHSEGAHPA	5(R)	trace
2	MYSFINDHSEGAHPR	40(R)	13.6
3	GTIYSR	53(S)	1.5
4	GAMLAR	39(R)	16.8

Reaction conditions: 0.05 of mmol p-nitrobenzaldehyde, 150 μL of solvent containing acetone/H_2_O 3/1 (*v*/*v*), and 4 mol% peptide were used for 5 days at room temperature while being agitated at 250 rpm in orbital shaker unless noted otherwise.

**Table 3:** The effect of different solvents on the asymmetric aldol reaction.

Entry	Solvent	ee%	Yield%
1	Acetone	18	7.1
2	Acetone/H_2_O (3:1)	40	13.6
3	Acetone/H_2_O (2:3)	34	1.9
4	MeOH	44	9.0
5	EtOH	38	5.7
6	*i*-PrOH	29	6.0
7	DMSO/H_2_O (100:1)	73	1.8
8	DMF/H_2_O (100:1)	58	8.0
9	CH_3_CN	22	1.7
10	toluene	11	3.0
11	DCM	34	0.8
12	DCE	41	0.6
13	Et_2_O	23	9.2
14	n-hexane	22	5.8
15	pyridine	51	2.5
16	ethyl acetate	18	4.3

Reaction conditions: 0.05 mmol of p-nitrobenzaldehyde, 200 μL of acetone, 300 μL of solvent, and 4 mol% Peptide were used for 5 days at room temperature while being agitated at 250 rpm in orbital shaker unless noted otherwise.

**Table 4:** The effect of temperature on the asymmetric aldol reaction between acetone and p-nitrobenzaldehyde.

Entry	Temperature °C	ee%	Yield%
1	4	13	21.2
2	25	27	78.5
3	30	28	81.2
4	37	34	87.2
5	45	33	79.4

Reaction conditions: 0.05 mmol of p-nitrobenzaldehyde, 150 μL of solvent containing acetone/buffer solution 3/1 (*v*/*v*), and 12 mol% peptide were used for 5 days at room temperature while being agitated at 250 rpm in orbital shaker unless noted otherwise. Buffer solution: 50 mM Gly-NaOH solution at pH 9.0.

**Table 5:** The effect of solvent, peptide dosage, and temperature on the asymmetric aldol reaction between acetone and p-nitrobenzaldehyde in DMSO (organic solvent).

Entry	Solvent		Peptide Dosage%	Temperature (°C)	ee%	Yield%
	H_2_O or Buffer Solution (μL)	Acetone (μL)				
1	3	200	4	25	73	1.8
2	3	200	4	37	69	14.6
3	3	200	4	45	61	17.2
4	10	200	4	25	68	9.5
5	20	200	4	25	58	8.7
6	25	200	4	25	56	8.4
7	3	150	4	25	69	6.9
8	3	250	4	25	72	10.1
9	3	300	4	25	73	13.0
10	10	300	12	25	65	20.7
11	10	300	20	25	68	31.6
12	10	300	30	25	66	29.9
13	10	300	20	37	63	80.8

Reaction conditions: 0.05 mmol of p-nitrobenzaldehyde was reacted with acetone in a mixture of 300 μL of DMSO and two other solvents for 5 days at room temperature while being agitated at 250 rpm in an orbital shaker unless noted otherwise. Buffer solution: 50 mM Gly-NaOH solution at pH 9.0.

## Data Availability

Not applicable.

## Supplementary Materials

The following supporting information can be downloaded at: https://www.mdpi.com/article/10.3390/molecules28196985/s1. S1: The original sequence of pET-17b(+) plasmid; S2: Primers used in this study; S3: The resulting sequence of pET-17b(+)-LmrREKP2 plasmid; S4: Agarose gel electrophoresis analysis; S5: SDS-PAGE analysis; S6: MS analysis of peptide2; S7: Chiral HPLC analysis; S8: ^1^H NMR analysis; S9: Some pictures documenting the process of the experiment.

## Sample Availability

Samples are available from the authors.

## Abbreviations

Amp	ampicillin
Arg	arginine
D	aspartic acid
DCE	1,2-dichloroethane
DCM	dichloromethane
DMSO	dimethyl sulfoxide
*E. coli*	*Escherichia coli*
EK	enterokinase
Gly	glycine
His	histidine
IPTG	isopropyl-β-d-thiogalactoside
K	lysine
LB	Luria–Bertani
Ni-NTA	Nickel-nitrilotriacetic acid
P	peptide
PCR	Polymerase Chain Reaction
SDS-PAGE	sodium dodecyl sulfate−polyacrylamide gel electrophoresis

## References

[1] Soai K., Niwa S. (1992). Enantioselective Addition of Organozinc Reagents to Aldehydes. Chem. Rev..

[2] Li Y.-Y., Yu S.-L., Shen W.-Y., Gao J.-X. (2015). Iron-, Cobalt-, and Nickel-Catalyzed Asymmetric Transfer Hydrogenation and Asymmetric Hydrogenation of Ketones. Acc. Chem. Res..

[3] Yamashita Y., Yasukawa T., Yoo W.-J., Kitanosono T., Kobayashi S. (2018). Catalytic Enantioselective Aldol Reactions. Chem. Soc. Rev..

[4] Trost B.M., Brindle C.S. (2010). The Direct Catalytic Asymmetric Aldol Reaction. Chem. Soc. Rev..

[5] He Y.-H., Li H.-H., Chen Y.-L., Xue Y., Yuan Y., Guan Z. (2012). Chymopapain-Catalyzed Direct Asymmetric Aldol Reaction. Adv. Synth. Catal..

[6] Haoran W., Zhi W., Hong Z., Ge C., Hong Y., Lei W. (2014). Enzyme Catalytic Promiscuity: Asymmetric Aldol Addition Reaction Catalyzed by a Novel Thermophilic Esterase in Organic Solvent. Green Chem. Lett. Rev..

[7] Finn M.G., Lerner R.A., Barbas C.F. (1998). Cofactor-Induced Refinement of Catalytic Antibody Activity:  A Metal-Specific Allosteric Effect. J. Am. Chem. Soc..

[8] Xu Y., Yamamoto N., Janda K.D. (2004). Catalytic Antibodies: Hapten Design Strategies and Screening Methods. Bioorganic Med. Chem..

[9] List B. (2007). Introduction:  Organocatalysis. Chem. Rev..

[10] Dondoni A., Massi A. (2008). Asymmetric Organocatalysis: From Infancy to Adolescence. Angew. Chem. Int. Ed..

[11] Ashokkumar V., Chithiraikumar C., Siva A. (2016). Binaphthyl-Based Chiral Bifunctional Organocatalysts for Water Mediated Asymmetric List–Lerner–Barbas Aldol Reactions. Org. Biomol. Chem..

[12] Hughes D.L. (2022). Highlights of the Recent Patent Literature: Focus on Asymmetric Organocatalysis. Org. Process Res. Dev..

[13] Inoue H., Kikuchi M., Ito J., Nishiyama H. (2008). Chiral Phebox–Rhodium Complexes as Catalysts for Asymmetric Direct Aldol Reaction. Tetrahedron.

[14] Chen Y.-L., Li W., Liu Y., Guan Z., He Y.-H. (2013). Trypsin-Catalyzed Direct Asymmetric Aldol Reaction. J. Mol. Catal. B Enzym..

[15] List B., Lerner R.A., Barbas C.F. (1999). Enantioselective Aldol Cyclodehydrations Catalyzed by Antibody 38C2. Org. Lett..

[16] Zheng B.-L., Liu Q.-Z., Guo C.-S., Wang X.-L., He L. (2007). Highly Enantioselective Direct Aldol Reaction Catalyzed by Cinchona Derived Primary Amines. Org. Biomol. Chem..

[17] Bastida D., Liu Y., Tian X., Escudero-Adán E., Melchiorre P. (2013). Asymmetric Vinylogous Aldol Reaction via H-Bond-Directing Dienamine Catalysis. Org. Lett..

[18] List B., Lerner R.A., Barbas C.F. (2000). Proline-Catalyzed Direct Asymmetric Aldol Reactions. J. Am. Chem. Soc..

[19] Córdova A., Zou W., Dziedzic P., Ibrahem I., Reyes E., Xu Y. (2006). Direct Asymmetric Intermolecular Aldol Reactions Catalyzed by Amino Acids and Small Peptides. Chem. A Eur. J..

[20] Kofoed J., Nielsen J., Reymond J.-L. (2003). Discovery of New Peptide-Based Catalysts for the Direct Asymmetric Aldol Reaction. Bioorganic Med. Chem. Lett..

[21] Wennemers H. (2011). Asymmetric Catalysis with Peptides. Chem. Commun..

[22] Córdova A., Notz W., Barbas C.F. (2002). Direct Organocatalytic Aldol Reactions in Buffered Aqueous Media. Chem. Commun..

[23] Tang Z., Yang Z.-H., Chen X.-H., Cun L.-F., Mi A.-Q., Jiang Y.-Z., Gong L.-Z. (2005). A Highly Efficient Organocatalyst for Direct Aldol Reactions of Ketones with Aldedydes. J. Am. Chem. Soc..

[24] Mase N., Nakai Y., Ohara N., Yoda H., Takabe K., Tanaka F., Barbas C.F. (2006). Organocatalytic Direct Asymmetric Aldol Reactions in Water. J. Am. Chem. Soc..

[25] Huang W., Tian H., Xu H., Zheng L., Liu Q., Zhang S. (2011). L-Valine Dipeptide Organocatalysts with Two Amide Units for the Direct Asymmetric Aldol Reaction in Brine. Catal. Lett..

[26] Wang Y., Wang Y., Liu L., Sang K., Zhang C., Satoh T. (2023). Asymmetric Aldol Reaction Catalyzed by Amino Acid Tetrapeptides (L-Pro-L-Pro-L-Phe-L-Phe-OMe). React. Chem. Eng..

[27] Al-Momani L.A., Lang H., Lüdeke S. (2023). A Pathway for Aldol Additions Catalyzed by L-Hydroxyproline-Peptides via a β-Hydroxyketone Hemiaminal Intermediate. Chemistry.

[28] Bayat S., Tejo B.A., Abdulmalek E., Salleh A.B., Normi Y.M., Abdul Rahman M.B. (2014). Rational Design of Mimetic Peptides Based on Aldo-Ketoreductase Enzyme as Asymmetric Organocatalysts in Aldol Reactions. RSC Adv..

[29] Peme T., Brady D., Juma W., Makatini M. (2021). Development of Fructose-1,6-Bisphosphate Aldolase Enzyme Peptide Mimics as Biocatalysts in Direct Asymmetric Aldol Reactions. RSC Adv..

[30] Davie E.A.C., Mennen S.M., Xu Y., Miller S.J. (2007). Asymmetric Catalysis Mediated by Synthetic Peptides. Chem. Rev..

[31] Metrano A.J., Chinn A.J., Shugrue C.R., Stone E.A., Kim B., Miller S.J. (2020). Asymmetric Catalysis Mediated by Synthetic Peptides, Version 2.0: Expansion of Scope and Mechanisms. Chem. Rev..

[32] Lundberg H., Tinnis F., Selander N., Adolfsson H. (2014). Catalytic Amide Formation from Non-Activated Carboxylic Acids and Amines. Chem. Soc. Rev..

[33] Krattiger P., Kovasy R., Revell J.D., Ivan S., Wennemers H. (2005). Increased Structural Complexity Leads to Higher Activity: Peptides as Efficient and Versatile Catalysts for Asymmetric Aldol Reactions. Org. Lett..

[34] Wieczorek R., Adamala K., Gasperi T., Polticelli F., Stano P. (2017). Small and Random Peptides: An Unexplored Reservoir of Potentially Functional Primitive Organocatalysts. The Case of Seryl-Histidine. Life.

[35] Carvalho S., Peralta Reis D.Q., Pereira S.V., Kalafatovic D., Pina A.S. (2022). Catalytic Peptides: The Challenge between Simplicity and Functionality. Isr. J. Chem..

[36] Schnitzer T., Wiesner M., Krattiger P., Revell J.D., Wennemers H. (2017). Is More Better? A Comparison of Tri- and Tetrapeptidic Catalysts. Org. Biomol. Chem..

[37] Dryland A., Sheppard R.C. (1986). Peptide Synthesis. Part 8. A System for Solid-Phase Synthesis under Low Pressure Continuous Flow Conditions. J. Chem. Soc. Perkin Trans..

[38] Gao Y., Zhao F., Wang Q., Zhang Y., Xu B. (2010). Small Peptide Nanofibers as the Matrices of Molecular Hydrogels for Mimicking Enzymes and Enhancing the Activity of Enzymes. Chem. Soc. Rev..

[39] Isidro-Llobet A., Kenworthy M.N., Mukherjee S., Kopach M.E., Wegner K., Gallou F., Smith A.G., Roschangar F. (2019). Sustainability Challenges in Peptide Synthesis and Purification: From R&D to Production. J. Org. Chem..

[40] Deng T., Ge H., He H., Liu Y., Zhai C., Feng L., Yi L. (2017). The Heterologous Expression Strategies of Antimicrobial Peptides in Microbial Systems. Protein Expr. Purif..

[41] Issaq H.J., Conrads T.P., Janini G.M., Veenstra T.D. (2002). Methods for Fractionation, Separation and Profiling of Proteins and Peptides. Electrophoresis.

[42] Spinck M., Piedrafita C., Robertson W.E., Elliott T.S., Cervettini D., De La Torre D., Chin J.W. (2023). Genetically Programmed Cell-Based Synthesis of Non-Natural Peptide and Depsipeptide Macrocycles. Nat. Chem..

[43] Kaguchi R., Katsuyama A., Sato T., Takahashi S., Horiuchi M., Yokota S., Ichikawa S. (2023). Discovery of Biologically Optimized Polymyxin Derivatives Facilitated by Peptide Scanning and In Situ Screening Chemistry. J. Am. Chem. Soc..

[44] Zheng W., Pu Z., Xiao L., Xu G., Yang L., Yu H., Wu J. (2022). Mutability-Landscape-Guided Engineering of l-Threonine Aldolase Revealing the Prelog Rule in Mediating Diastereoselectivity of C-C Bond Formation. Angew. Chem. Int. Ed..

