# Supplementary material for: A Simple Screening and Optimization Bioprocess for Long-Chain Peptide Catalysts Applied to Asymmetric Aldol Reaction
Source: Molecules. 2023 Oct 9;28(19):6985. doi: 10.3390/molecules28196985 (PMC10574572; doi:10.3390/molecules28196985)

A Simple Screening and Optimization Bioprocess for Long-Chain Peptide Catalysts Applied to Asymmetric Aldol Reaction

Shulin Wang, Haidong Teng, Lan Wang, Pengcheng Li, Xinghao Yuan, Xi Sang, Jianping Wu, Lirong Yang and Gang Xu *

College of Chemical and Biological Engineering, Zhejiang University, Hangzhou 310058, China
* Correspondence: xugang_1030@zju.edu.cn

Supporting Information

Table of Contents

S1. The original sequence of pET-17b(+) plasmid 2
S2. Primers used in this study 3
S3. The resulting sequence of pET-17b(+)-LmrREKP2 plasmid 3
S4. Agarose gel electrophoresis analysis 4
S5. SDS-PAGE analysis 5
S6. MS analysis of peptide2 5
S7. Chiral HPLC analysis 5
S8. ^{1}H NMR analysis 8
S9. Some pictures documenting the process of the experiment. 8

S1. The original sequence of pET-17b(+) plasmid

acgccgggcaagagcaactcggtcgccgcatacactattctcagaatgacttggttgagtactcaccagtcacagaaaagcatcttacggatg
gcatgacagtaagagaattatgcagtgctgccataaccatgagtgataacactgcggccaacttacttctgacaacgatcggaggaccgaag
gagctaaccgcttttttgcacaacatgggggatcatgtaactcgccttgatcgttgggaaccggagctgaatgaagccataccaaacgacgag
cgtgacaccacgatgcctgcagcaatggcaacaacgttgcgcaaactattaactggcgaactacttactctagcttcccggcaacaattaatag
actggatggaggcggataaagttgcaggaccacttctgcgctcggcccttccggctggctggtttattgctgataaatctggagccggtgagc
gtgggtctcgcggtatcattgcagcactggggccagatggtaagccctcccgtatcgtagttatctacacgacggggagtcaggcaactatgg
atgaacgaaatagacagatcgctgagataggtgcctcactgattaagcattggtaactgtcagaccaagtttactcatatatactttagattgattt
aaaacttcatttttaatttaaaaggatctaggtgaagatcctttttgataatctcatgaccaaaatcccttaacgtgagttttcgttccactgagcgtca
gaccccgtagaaaagatcaaaggatcttcttgagatcctttttttctgcgcgtaatctgctgcttgcaaacaaaaaaaccaccgctaccagcggt
ggtttgtttgccggatcaagagctaccaactctttttccgaaggtaactggcttcagcagagcgcagataccaaatactgtccttctagtgtagcc
gtagttaggccaccacttcaagaactctgtagcaccgcctacatacctcgctctgctaatcctgttaccagtggctgctgccagtggcgataagt
cgtgtcttaccgggttggactcaagacgatagttaccggataaggcgcagcggtcgggctgaacggggggttcgtgcacacagcccagctt
ggagcgaacgacctacaccgaactgagatacctacagcgtgagctatgagaaagcgccacgcttcccgaagggagaaaggcggacaggt
atccggtaagcggcagggtcggaacaggagagcgcacgagggagcttccagggggaaacgcctggtatctttatagtcctgtcgggtttcg
ccacctctgacttgagcgtcgatttttgtgatgctcgtcaggggggcggagcctatggaaaaacgccagcaacgcggcctttttacggttcctg
gccttttgctggccttttgctcacatgttctttcctgcgttatcccctgattctgtggataaccgtattaccgcctttgagtgagctgataccgctcgc
cgcagccgaacgaccgagcgcagcgagtcagtgagcgaggaagcggaagagcgcctgatgcggtattttctccttacgcatctgtgcggta
tttcacaccgcatatatggtgcactctcagtacaatctgctctgatgccgcatagttaagccagtatacactccgctatcgctacgtgactgggtc
atggctgcgccccgacacccgccaacacccgctgacgcgccctgacgggcttgtctgctcccggcatccgcttacagacaagctgtgaccg
tctccgggagctgcatgtgtcagaggttttcaccgtcatcaccgaaacgcgcgaggcagctgcggtaaagctcatcagcgtggtcgtgaagc
gattcacagatgtctgcctgttcatccgcgtccagctcgttgagtttctccagaagcgttaatgtctggcttctgataaagcgggccatgttaagg
gcggtttttttcctgtttggtcactgatgcctccgtgtaagggggatttctgttcatgggggtaatgataccgatgaaacgagagaggatgctcacg
atacgggttactgatgatgaacatgcccggttactggaacgttgtgagggtaaacaactggcggtatggatgcggcgggaccagagaaaaat
cactcagggtcaatgccagcgcttcgttaatacagatgtaggtgttccacagggtagccagcagcatcctgcgatgcagatccggaacataat
ggtgcagggcgctgacttccgcgtttccagactttacgaaacacggaaaccgaagaccattcatgttgttgctcaggtcgcagacgttttgcag
cagcagtcgcttcacgttcgctcgcgtatcggtgattcattctgctaaccagtaaggcaaccccgccagcctagccgggtcctcaacgacagg
agcacgatcatgcgcacccgtggccaggacccaacgctgcccgagatctcgatcccgcgaaattaatacgactcactatagggagaccaca
acggtttccctctagaaataattttgtttaactttaagaaggagatatacatatggctagcatgactggtggacagcaaatgggtcgggattcaag
cttCATGGGTGCCGAAATCCCGAAAGAAATGCTGCGTGCTCAAACCAATGTCATCCTGC
TGAATGTCCTGAAACAAGGCGATAACTATGTGTATGGCATTATCAAACAGGTGAAAG
AAGCGAGCAACGGTGAAATGGAACTGAATGAAGCCACCCTGTATACGATTTTTGATC
GTCTGGAACAGGACGGCATTATCAGCTCTTACTGGGGTGATGAAAGTCAAGGCGGTC
GTCGCAAATATTACCGTCTGACCGAAATCGGCCATGAAAACATGCGCCTGGCGTTCG
AATCCTGGAGTCGTGTGGACAAAATCATTGAAAATCTGGAAGCAAACAAAAAATCTG
AAGCGATCAAATCTAGAGGTGGCAGCGGTGGCCATCACCACCACCACCACTGAg
gatccactagtaacggccgccagtgtgctggaattctgcagatatccatcacactggcggccgctcgagcagatccggctgctaacaaagcc
cgaaaggaagctgagttggctgctgccaccgctgagcaataactagcataaccccttggggcctctaaacgggtcttgaggggttttttgctga
aaggaggaactatatccggataattcttgaagacgaaagggcctcgtgatacgcctatttttataggttaatgtcatgataataatggtttcttaga
cgtcaggtggcacttttcggggaaatgtgcgcggaacccctatttgtttatttttctaaatacattcaaatatgtatccgctcatgagacaataaccc
tgataaatgcttcaataatattgaaaaaggaagagtatgagtattcaacatttccgtgtcgcccttattcccttttttgcggcattttgccttcctgtttttt
gctcacccagaaacgctggtgaaagtaaaagatgctgaagatcagttgggtgcacgagtgggttacatcgaactggatctcaacagcggtaa
gatccttgagagttttcgccccgaagaacgttttccaatgatgagcacttttaaagttctgctatgtggcgcggtattatcccgtgttg

The sequence corresponding to the **UPPERCASE LETTERS** represents the LmrR protein.

S2. Primers used in this study

Name	Primers
EKF	GACGACGACGACAAGCATCACCACCACCACCACCA
EKR	TGCTTGTCGTCGTCGTCGCCACCGCTGCCACCTCT
P1F	TTAGCAACAGCACGGAACGTCATCACCACCACCACCACCA
P1R	GTTCCGTGCTGTTGCTAATATAGACCAGCTTGTCGTCGTCGTCGCC
P2F	caacgatcacagcgaaggtgcacatccgcgtCATCACCACCACCACCACCA
P2R	cttcgctgtgatcgttgttgaaggagtacatCTTGTCGTCGTCGTCGCC
P3F	GGTACCATCTATTCCAAACATCACCACCACCACCACCA
P3R	TTTGGAATAGATGGTACCCTTGTCGTCGTCGTCGCC
P4F	GGCGCAATGCTGGCAAAACATCACCACCACCACCACCA
P4R	TTTTGCCAGCATTGCGCCCTTGTCGTCGTCGTCGCC
StopF	ATCCGCGTtgaCACCACCACCACCACCACTGA
StopR	GTGGTGtcaACGCGGATGTGCACCTTCGCTGT
NHisF	caccaccaccaccaccacGGTGCCGAAATCCCGAAA
NHisR	gtggtggtggtggtggtgCATGAAGCTTGAATCCCGACC
P2N5?F	CTCCTTCNNNAACGATCACAGCGAAGGTGCAC
P2N5?R	GATCGTTNNNGAAGGAGTACATCTTGTCGTCGTCG
P2N6?F	CCTTCAACNNNGATCACAGCGAAGGTGCACAT
P2N6?R	GTGATCNNNGTTGAAGGAGTACATCTTGTCGTCG
P2H8?F	CAACGATNNNAGCGAAGGTGCACATCCGCGTT
P2H8?R	CTTCGCTNNNATCGTTGTTGAAGGAGTACATCTTGT
P2R15AF	TCATCCGgcaTGACACCACCACCACCACCACT
P2R15AR	GGTGTCAtgcCGGATGAGCACCCTCAGAGTGG
P3K6RF	ATCTACAGCcgcTGACACCACCACCACCACCA
P3K6RR	TGTCAgcgGCTGTAGATCGTGCCCTTGTCGTC
P4K6RF	ATGCTGGCAcgtTGACACCACCACCACCACCA
P4K6RR	TGTCAacgTGCCAGCATGGCGCCCTTGTCGTC

S3. The resulting sequence of pET-17b(+)-LmrREKP2 plasmid

acgccgggcaagagcaactcggtcgccgcatacactattctcagaatgacttggttgagtactcaccagtcacagaaaagcatcttacggatg
gcatgacagtaagagaattatgcagtgctgccataaccatgagtgataacactgcggccaacttacttctgacaacgatcggaggaccgaag
gagctaaccgcttttttgcacaacatgggggatcatgtaactcgccttgatcgttgggaaccggagctgaatgaagccataccaaacgacgag
cgtgacaccacgatgcctgcagcaatggcaacaacgttgcgcaaactattaactggcgaactacttactctagcttcccggcaacaattaatag
actggatggaggcggataaagttgcaggaccacttctgcgctcggcccttccggctggctggtttattgctgataaatctggagccggtgagc
gtgggtctcgcggtatcattgcagcactggggccagatggtaagccctcccgtatcgtagttatctacacgacggggagtcaggcaactatgg
atgaacgaaatagacagatcgctgagataggtgcctcactgattaagcattggtaactgtcagaccaagtttactcatatatactttagattgattt
aaaacttcatttttaatttaaaaggatctaggtgaagatcctttttgataatctcatgaccaaaatcccttaacgtgagttttcgttccactgagcgtca
gaccccgtagaaaagatcaaaggatcttcttgagatcctttttttctgcgcgtaatctgctgcttgcaaacaaaaaaaccaccgctaccagcggt
ggtttgtttgccggatcaagagctaccaactctttttccgaaggtaactggcttcagcagagcgcagataccaaatactgtccttctagtgtagcc
gtagttaggccaccacttcaagaactctgtagcaccgcctacatacctcgctctgctaatcctgttaccagtggctgctgccagtggcgataagt
cgtgtcttaccgggttggactcaagacgatagttaccggataaggcgcagcggtcgggctgaacggggggttcgtgcacacagcccagctt
ggagcgaacgacctacaccgaactgagatacctacagcgtgagctatgagaaagcgccacgcttcccgaagggagaaaggcggacaggt
atccggtaagcggcagggtcggaacaggagagcgcacgagggagcttccagggggaaacgcctggtatctttatagtcctgtcgggtttcg

ccacctctgacttgagcgtcgatttttgtgatgctcgtcaggggggcggagcctatggaaaaacgccagcaacgcggcctttttacggttcctg
gccttttgctggccttttgctcacatgttctttcctgcgttatcccctgattctgtggataaccgtattaccgcctttgagtgagctgataccgctcgc
cgcagccgaacgaccgagcgcagcgagtcagtgagcgaggaagcggaagagcgcctgatgcggtattttctccttacgcatctgtgcggta
tttcacaccgcatatatggtgcactctcagtacaatctgctctgatgccgcatagttaagccagtatacactccgctatcgctacgtgactgggtc
atggctgcgccccgacacccgccaacacccgctgacgcgccctgacgggcttgtctgctcccggcatccgcttacagacaagctgtgaccg
tctccgggagctgcatgtgtcagaggttttcaccgtcatcaccgaaacgcgcgaggcagctgcggtaaagctcatcagcgtggtcgtgaagc
gattcacagatgtctgcctgttcatccgcgtccagctcgttgagtttctccagaagcgttaatgtctggcttctgataaagcgggccatgttaagg
gcggtttttcctgtttggtcactgatgcctccgtgtaagggggatttctgttcatgggggtaatgataccgatgaaacgagagaggatgctcacg
atacgggttactgatgatgaacatgcccggttactggaacgttgtgagggtaaacaactggcggtatggatgcggcgggaccagagaaaaat
cactcagggtcaatgccagcgcttcgttaatacagatgtaggtgttccacagggtagccagcagcatcctgcgatgcagatccggaacataat
ggtgcagggcgctgacttccgcgtttccagactttacgaaacacggaaaccgaagaccattcatgttgttgctcaggtcgcagacgttttgcag
cagcagtcgcttcacgttcgctcgcgtatcggtgattcattctgctaaccagtaaggcaaccccgccagcctagccgggtcctcaacgacagg
agcacgatcatgcgcacccgtggccaggacccaacgctgcccgagatctcgatcccgcgaaattaatacgactcactatagggagaccaca
acggtttccctctagaaataattttgtttaactttaagaaggagatatacatatggctagcatgactggtggacagcaaatgggtcgggattcaag
cttCATGCACCACCACCACCACCACGGTGCCGAAATCCCGAAAGAAATGCTGCGTGCTC
AAACCAATGTCATCCTGCTGAATGTCCTGAAACAAGGCGATAACTATGTGTATGGCA
TTATCAAACAGGTGAAAGAAGCGAGCAACGGTGAAATGGAACTGAATGAAGCCACC
CTGTATACGATTTTTGATCGTCTGGAACAGGACGGCATTATCAGCTCTTACTGGGGTG
ATGAAAGTCAAGGCGGTCGTCGCAAATATTACCGTCTGACCGAAATCGGCCATGAAA
ACATGCGCCTGGCGTTCGAATCCTGGAGTCGTGTGGACAAAATCATTGAAAATCTGG
AAGCAAACAAAAAATCTGAAGCGATCAAATCTAGAGGTGGCAGCGGTGGCGACGAC
GACGACAAGATGTACAGCTTCAACAACGACCATAGCGAAGGTGCACATCCTCGTtgaca
ccaccaccaccaccactgaggatccactagtaacggccgccagtgtgctggaattctgcagatatccatcacactggcggccgctcgagca
gatccggctgctaacaaagcccgaaaggaagctgagttggctgctgccaccgctgagcaataactagcataaccccttggggcctctaaacg
ggtcttgaggggttttttgctgaaaggaggaactatatccggataattcttgaagacgaaagggcctcgtgatacgcctatttttataggttaatgt
catgataataatggtttcttagacgtcaggtggcacttttcggggaaatgtgcgcggaacccctatttgtttatttttctaaatacattcaaatatgtat
ccgctcatgagacaataaccctgataaatgcttcaataatattgaaaaaggaagagtatgagtattcaacatttccgtgtcgcccttattcccttttt
gcggcattttgccttcctgtttttgctcacccagaaacgctggtgaaagtaaaagatgctgaagatcagttgggtgcacgagtgggttacatcga
actggatctcaacagcggtaagatccttgagagttttcgccccgaagaacgttttccaatgatgagcacttttaaagttctgctatgtggcgcggt
attatcccgtgttg

The sequence corresponding to the **UPPERCASE LETTERS** represents the LmrREKP2 protein.

S4. Agarose gel electrophoresis analysis

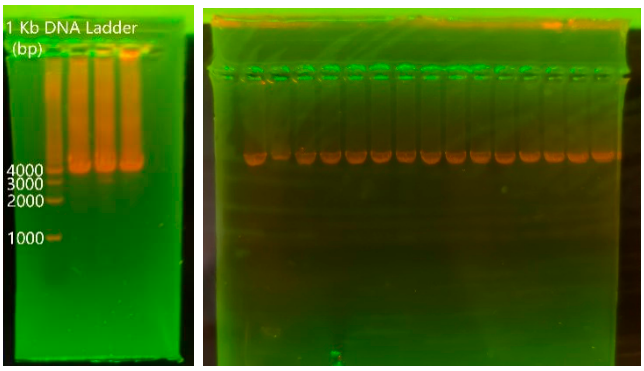

(a) The original and final plasmid results (b) Different mutated plasmids after saturation mutation

S5. SDS-PAGE analysis

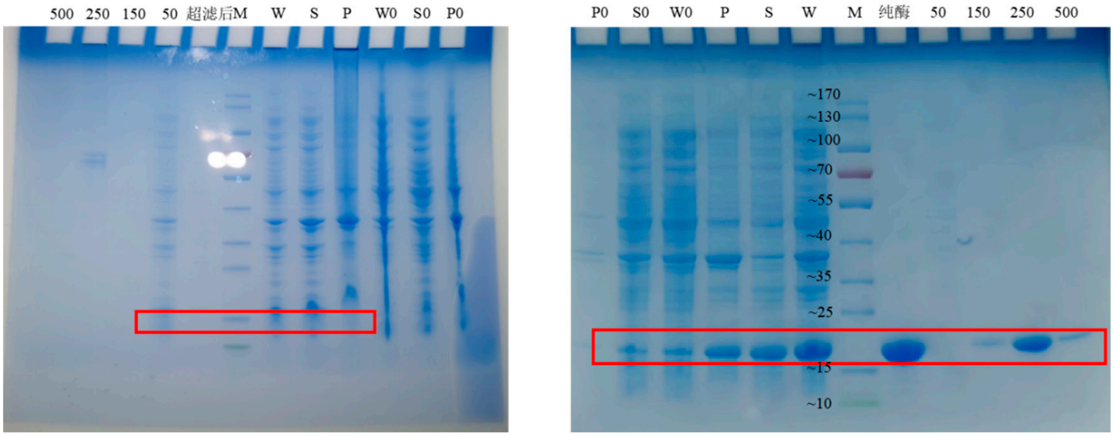

(a) C-terminus His-tagged fusion protein (b) N-terminus His-tagged fusion protein

M: Marker W: Whole-Cell Lysate P: Precipitate S: Supernatant 0: uninduced cell

50/150/250/500: 50/150/250/500 mM Imidazole buffer solution

超滤后:After ultrafiltration concentration 纯酶: pure enzyme

C-terminus His-tagged fusion protein could not be absorbed onto the Ni-NTA column, we introduced a new His-tag at the N-terminus of the original LmrR protein and removed His-tag from the C-terminus.

S6. MS analysis of peptide2

M.W :1761.75

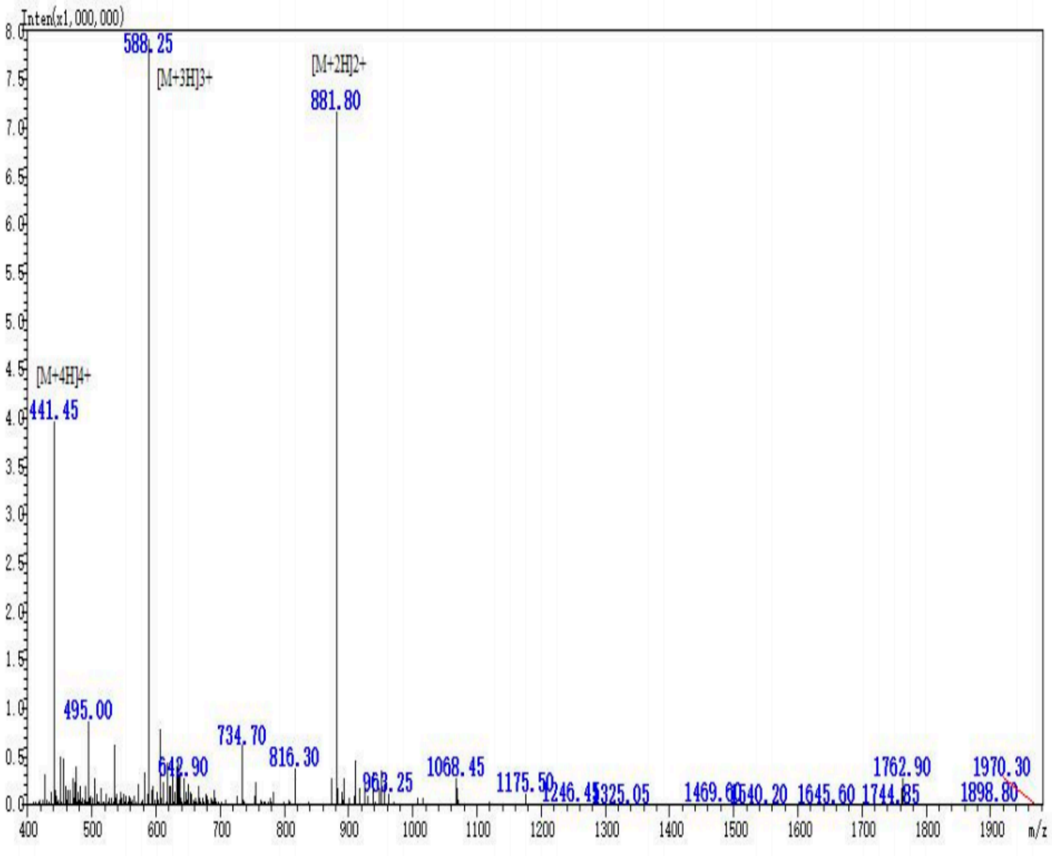

S7. Chiral HPLC analysis

The enantiomeric excess was determined by HPLC (Daicel Chiralpak AD-H, hexane/isopropanol =

90:10, flow rate 1.2 mL/min, λ = 254 nm), t_{Rmajor} = 12.955 min, t_{Rminor} = 13.499 min (hexane/isopropanol = 95:5, t_{Rmajor} = 25.518 min, t_{Rminor} = 26.719 min).(Our chiral column has been used for a long time, and we have observed fluctuations in its performance, with retention times differing across batches.)

S7.1 aldol reaction product racemer

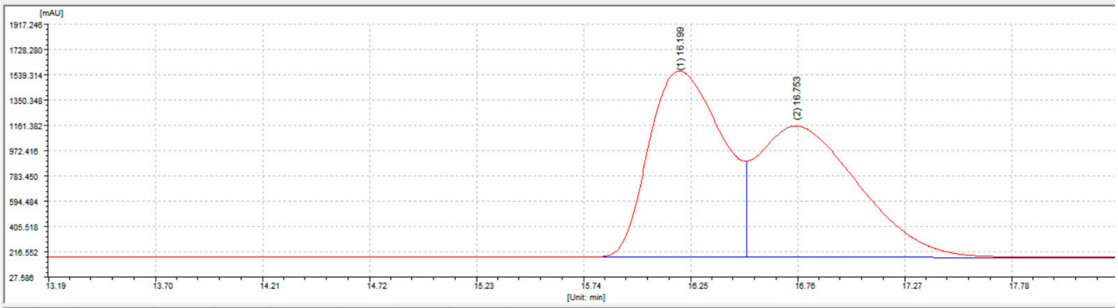

峰序	组分名	保留时间[min]	半峰宽[min]	峰高[uAU]	峰面积[uA...	峰面积[%]	含量[%]	峰类型
1		16.199	0.482	1392668.9	34098947.8	50.7476	50.7476	BV
2		16.753	0.567	982773.9	33094292.0	49.2524	49.2524	VB
			总计:	2375442.9	67193239.7	100.0000	100.0000	

From left to right are: peak order, component name, retention time(min), half-peak height(uAU), peak height(uAU), peak area (uAU), peak area (%), content (%), and peak type.

S7.2 peptide MYSFNNDHSEGAHPR powder asymmetric catalysis(hexane/isopropanol = 95:5)

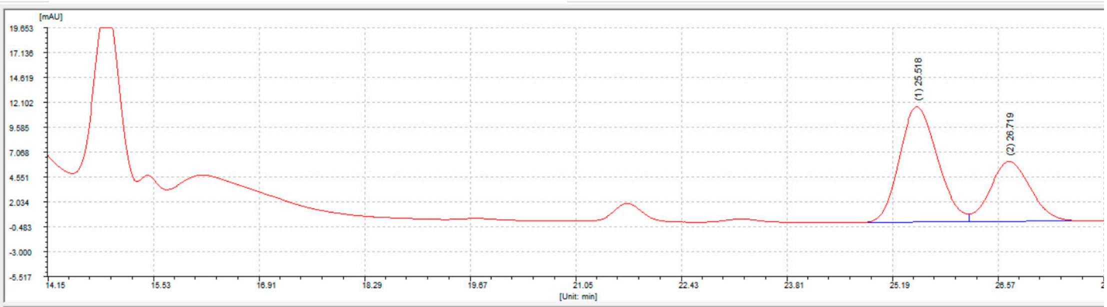

峰序	组分名	保留时间[min]	半峰宽[min]	峰高[uAU]	峰面积[uA...	峰面积[%]	含量[%]	峰类型
1		25.518	0.544	11712.8	407790.5	64.5568	64.5568	BV
2		26.719	0.574	6088.9	223886.7	35.4432	35.4432	VB
			总计:	17801.7	631677.2	100.0000	100.0000	

From left to right are: peak order, component name, retention time(min), half-peak height(uAU), peak height(uAU), peak area (uAU), peak area (%), content (%), and peak type.

S7.3 peptide MYSFINDHSEGAHPR asymmetric catalysis

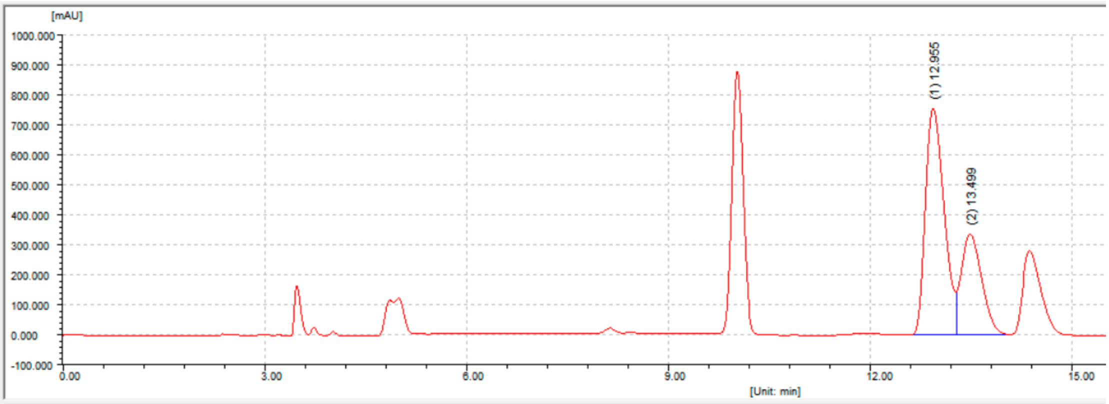

峰序	组分名	保留时间[min]	半峰宽[min]	峰高[uAU]	峰面积[uA...	峰面积[%]	含量[%]	峰类型
1		12.955	0.306	758224.9	14522692.6	66.8046	66.8046	BV
2		13.499	0.353	338156.7	7216363.2	33.1954	33.1954	VB
			总计:	1096381.6	21739055.8	100.0000	100.0000	

From left to right are: peak order, component name, retention time(min), half-peak height(uAU), peak height(uAU), peak area (uAU), peak area (%), content (%), and peak type.

S7.4 peptide MYSFINDHSEGAHPR asymmetric catalysis in DMSO

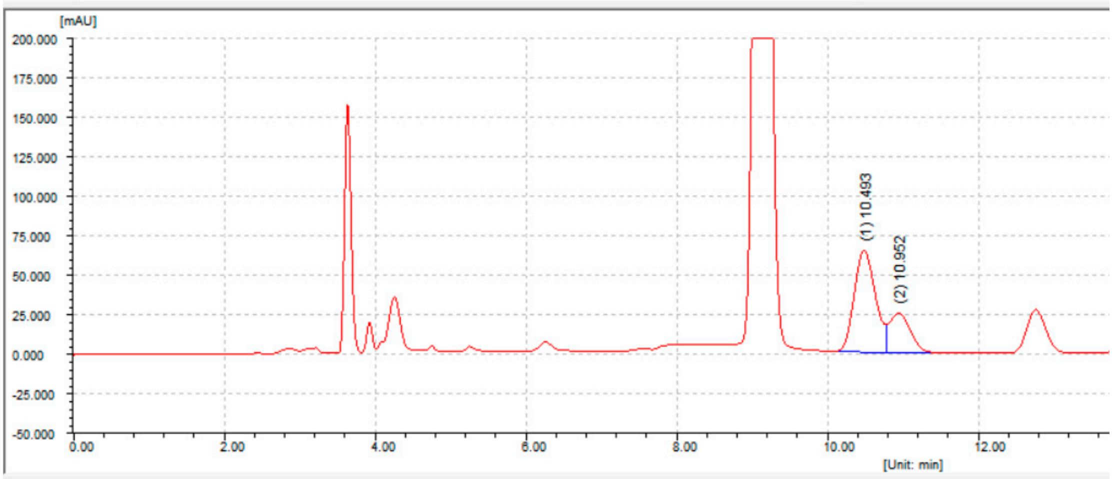

峰序	组分名	保留时间[min]	半峰宽[min]	峰高[uAU]	峰面积[uA...	峰面积[%]	含量[%]	峰类型
1		10.493	0.312	64421.2	1269033.5	72.0350	72.0350	BV
2		10.952	0.341	24881.7	492655.3	27.9649	27.9649	VB
			总计:	89303.0	1761688.8	100.0000	100.0000	

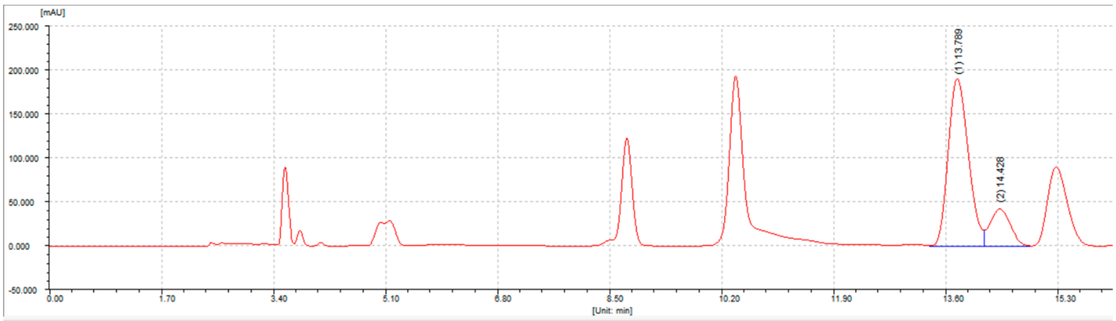

峰序	组分名	保留时间[min]	半峰宽[min]	峰高[uAU]	峰面积[uA...	峰面积[%]	含量[%]	峰类型
1		13.789	0.331	191236.7	4035215.1	81.4268	81.4268	VV
2		14.428	0.365	42409.3	920421.3	18.5732	18.5732	VV
			总计:	233646.0	4955636.4	100.0000	100.0000	

(Over 1 week)

From left to right are: peak order, component name, retention time(min), half-peak height(uAU), peak height(uAU), peak area (uAU), peak area (%), content (%), and peak type.

S8. ^{1}H NMR analysis

4-hydroxy-4-(4-nitrophenyl) butan-2-one

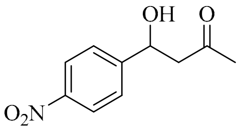

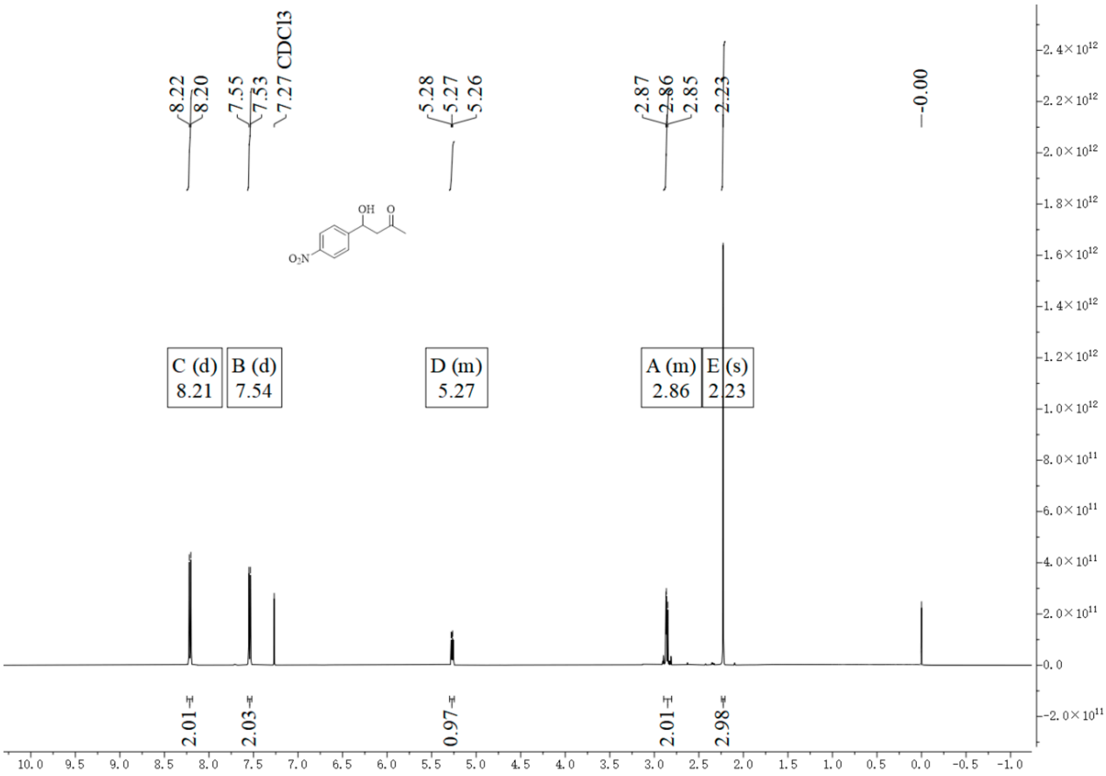

^{1}H NMR (500 MHz, CDCl3): δ 8.21 (d, J = 8.7 Hz, 2H), 7.54 (d, J = 8.3 Hz, 2H), 5.30 – 5.25 (m, 1H), 2.89 – 2.80 (m, 2H), 2.23 (s, 3H).

S9. Some pictures documenting the process of the experiment.

S9.1 Fusion-protein precipitated by ammonium sulfate(yellow-white solid)

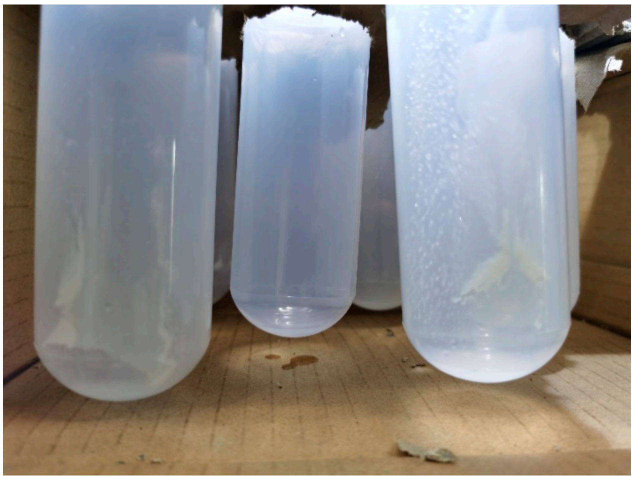

S9.2 Peptide powder before and after desalting (both freeze-dried)

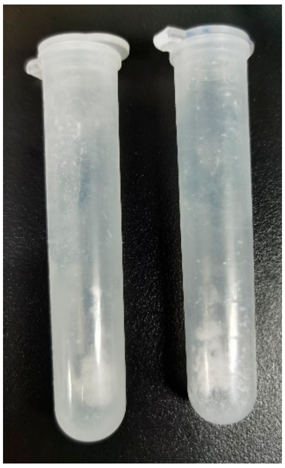

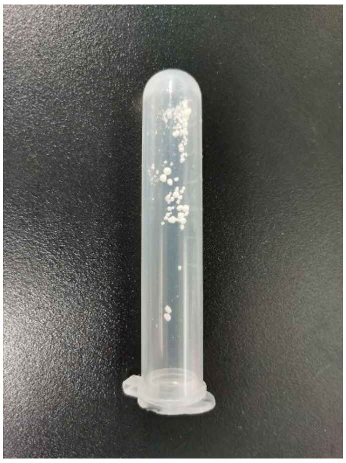

Supplement: Supplementary file 1 [file molecules-28-06985-s001.zip › molecules-2641859-supplementary.pdf]
